# Tear Cytokine Signature in Vernal Keratoconjunctivitis: A Chemokine-Remodeling Axis Associated with Disease Severity

**DOI:** 10.3390/ijms27167180

**Published:** 2026-08-11

**Authors:** Kartik Goel, Mehak Sapra, Prisha Warikoo, Shailja Tibrewal, Hirak Patra, Virender Singh Sangwan, Abha Gour, Anil Tiwari

**Affiliations:** 1 Eicher Shroff Centre for Stem Cell Research, Dr. Shroff’s Charity Eye Hospital, New Delhi 110002, India; kartikgoel02.1997@gmail.com (K.G.);; 2 Department of Ocular Genetics and Centre for Unknown and Rare Eye Diseases, Dr. Shroff’s Charity Eye Hospital, New Delhi 110002, India; 3 College of Pharmacy, University of Arkansas for Medical Sciences, Little Rock, AR 72205, USA; 4 Department of Pediatric Ophthalmology, Strabismus and Neuro-Ophthalmology, Dr. Shroff’s Charity Eye Hospital, New Delhi 110002, India; 5 Department of Surgical Biotechnology, Division of Surgery and Interventional Science, University College London, London NW3 2PS, UK; 6 Department of Cornea and Anterior Segment Services, Dr. Shroff’s Charity Eye Hospital, New Delhi 110002, India

**Keywords:** cytokines, ocular allergy, tear proteomics, vernal keratoconjunctivitis

## Abstract

Vernal keratoconjunctivitis (VKC) is a chronic pediatric ocular allergy that can progress from seasonal to persistent inflammation with vision-threatening complications. Although Th2-associated mechanisms have been implicated, the immune correlates of disease severity are not well defined. Tear samples from VKC patients (moderate intermittent and moderate persistent) and healthy controls were collected using Schirmer’s strips. Cytokine profiling was performed using the OLINK^®^ Target 48 Cytokine Panel. Differential expression, correlation with clinical features, and pathway enrichment analyses were performed. Compared to control, IL-15, CXCL11, CXCL9, MMP12, and CCL13 were significantly elevated in VKC, with higher levels in the persistent phenotype. These cytokines correlated with symptom duration, limbal involvement, and papillary hypertrophy. Pathway analysis revealed enrichment of IL-17, JAK–STAT, and chemokine signaling pathways. VKC severity is associated with distinct tear cytokine signatures, with the persistent phenotype showing enhanced chronic inflammatory signaling. These findings identify candidate tear-based markers of disease severity that require validation in larger, independent, and longitudinal cohorts.

## 1. Introduction

Vernal keratoconjunctivitis (VKC) is a chronic, recurrent, and seasonally exacerbated allergic inflammation of the ocular surface that predominantly affects children and adolescents, particularly in warm, dry climates. The disease shows a higher prevalence in regions such as India, the Middle East, and Africa, with estimates suggesting it affects up to 3% of children in endemic zones [1,2,3]. Clinically, VKC is characterized by intense ocular itching, photophobia, tearing, mucous discharge, and the development of giant papillae on the upper tarsal conjunctiva or limbal hypertrophy. While many cases follow a seasonal and self-limiting course, a substantial subset of patients—particularly in Indian and Asian populations—progresses to a moderate persistent or severe phenotype marked by perennial inflammation, conjunctival hypertrophy, limbal remodeling, and sight-threatening complications including punctate epithelial erosions, shield ulcers, and corneal scarring. This chronic evolution is frequently associated with prolonged steroid exposure and significant morbidity, underscoring the need for a deeper mechanistic understanding of disease persistence [4,5,6,7,8,9,10,11]. The immunopathogenesis of VKC has classically been attributed to an IgE-mediated type I hypersensitivity reaction. Allergen exposure triggers mast cell degranulation with release of histamine, prostaglandins, and leukotrienes, leading to early-phase symptoms such as itching and conjunctival hyperaemia [1]. Beyond this acute phase, VKC has been widely regarded as a T-helper 2 (Th2)–driven disorder, with elevated levels of IL-4, IL-5, and IL-13 detected in tears and conjunctival tissues. These cytokines promote IgE class switching, eosinophil recruitment and survival, mucus hypersecretion, and epithelial remodeling [1,7,12,13,14,15,16,17,18,19]. A hallmark of VKC is the persistent infiltration of eosinophils into the conjunctiva, driven by chemokines such as eotaxin (CCL11) and RANTES (CCL5), with subsequent release of cytotoxic granule proteins including major basic protein and eosinophil cationic protein that disrupt epithelial integrity and contribute to corneal injury [12,20]. Total and allergen-specific IgE have also been detected in the tear fluid of patients with VKC, supporting the contribution of local ocular sensitization and mast-cell activation to disease pathogenesis [21]. Tear IgE may promote the release of histamine and Th2-associated cytokines and contribute to eosinophil recruitment, itching, conjunctival hyperaemia, and epithelial injury [21]. However, accumulating evidence indicates that this Th2-centric paradigm may incompletely capture the immunological complexity of chronic VKC. Studies have demonstrated increased expression of Th1-associated cytokines, including IFN-γ and IL-12, as well as Th17-related mediators such as IL-17 and IL-6, particularly in more severe or long-standing disease [1,11,12,16,21,22,23]. In parallel, conjunctival fibroblasts and epithelial cells respond to inflammatory cues by producing matrix metalloproteinases, including MMP1, MMP9, and MMP12, thereby driving extracellular matrix degradation, tissue remodeling, and conjunctival fibrosis—features that distinguish persistent VKC from intermittent disease. Together, these findings suggest that VKC represents a complex, multicellular immune disorder involving overlapping Th2, Th1, and Th17 signaling networks rather than a purely allergic condition. Despite these advances, clinical grading of VKC continues to rely predominantly on symptom-based systems such as the Bonini classification and the 5-5-5 exacerbation grading scale, which do not incorporate molecular biomarkers. Consequently, the immunological correlates that distinguish intermittent from persistent VKC—and that may predict disease chronicity or progression—remain poorly defined. Most mechanistic studies to date have relied on conjunctival biopsies, serum measurements, or low-plex tear assays, approaches limited by invasiveness, low sensitivity, and restricted molecular coverage. This gap has likely reinforced an oversimplified Th2-dominant view of VKC pathogenesis, particularly at the ocular surface where disease activity is most clinically relevant [11,19,24,25]. Tear fluid provides a minimally invasive and highly informative window into the local ocular immune environment, offering clear advantages over tissue biopsies, especially in pediatric populations [2,26,27]. Tears capture dynamic immune processes occurring at the ocular surface and are therefore well suited for biomarker discovery and disease stratification. Recent advances in high-sensitivity proteomic technologies, including the OLINK^®^ proximity extension assay (PEA), enable multiplexed detection of inflammatory mediators from small tear volumes. While conventional multiplex assays have been applied to VKC, few studies have employed OLINK-based tear cytokine profiling, and even fewer have stratified patients according to clinical severity or disease persistence [15,28,29]. In this study, we applied OLINK^®^ PEA-based tear proteomics [30] to comprehensively profile inflammatory cytokines in VKC patients stratified as moderate intermittent or moderate persistent based on the Bonini grading system. We hypothesized that (i) persistent VKC is associated with a distinct tear cytokine profile extending beyond classical Th2-associated mediators and (ii) tear cytokine levels correlate with clinical indicators of disease severity and chronicity. IL-4 detectability was evaluated as one component of the broader cytokine profile, while recognizing that the absence of detectable tear IL-4 does not exclude local IgE-dependent or Th2-associated mechanisms.

## 2. Results

### 2.1. Patient Demographics and Clinical Characteristics

**Figure 1:**
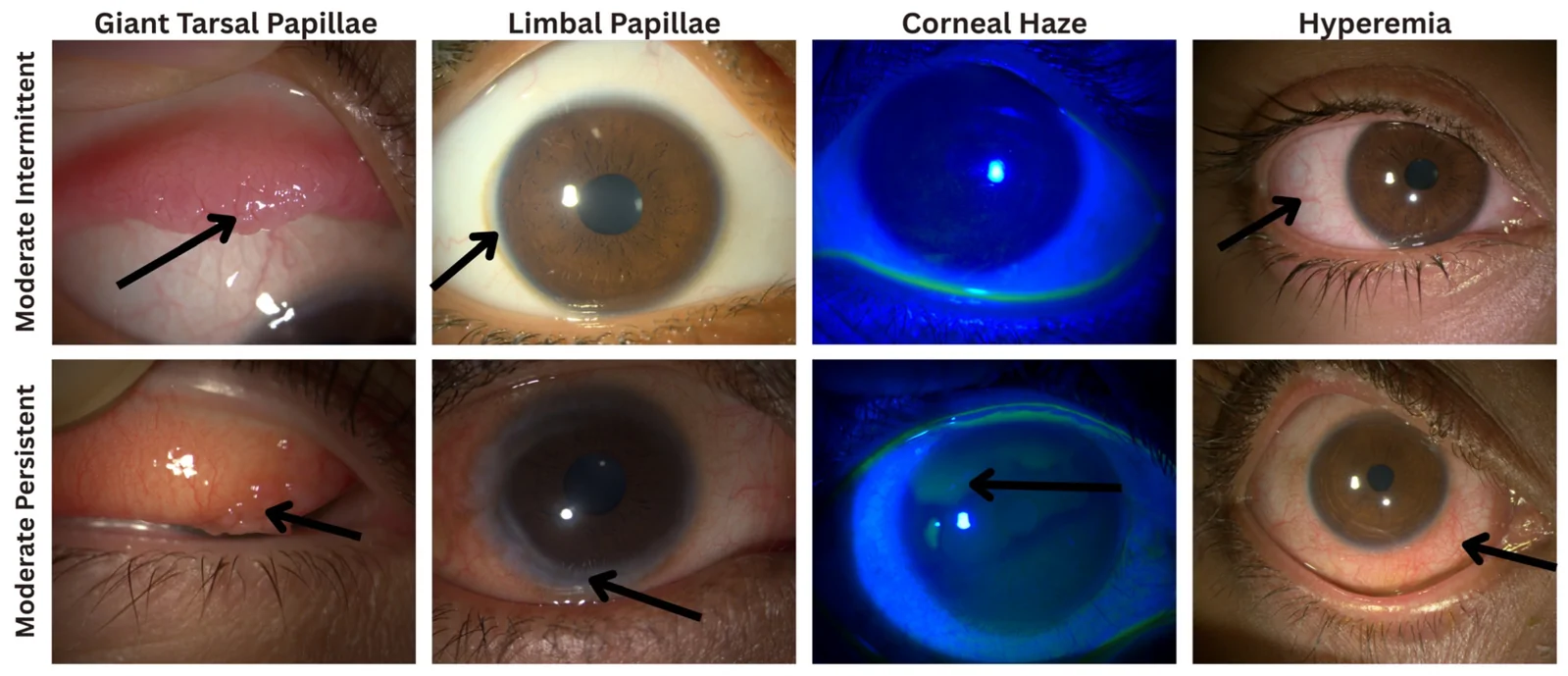
Representative clinical images demonstrating key ocular features in Moderate Intermittent and Moderate Persistent VKC. Moderate Intermittent VKC: Giant papillae are visible on the upper tarsal conjunctiva, accompanied by mild limbal papillae, localized corneal haze under fluorescein staining, and mild conjunctival hyperemia. Moderate Persistent VKC: More pronounced tarsal papillary hypertrophy is observed, along with prominent limbal thickening, extensive corneal epithelial damage and fluorescein uptake, and diffuse conjunctival hyperemia. Arrow are showing the clinical features written on top of of the respective figure.

Fifteen patients with clinically confirmed vernal keratoconjunctivitis (VKC) and ten healthy controls were included (Figure 1). The VKC cohort was predominantly male (80%), with a median age of 13 years (range: 8–27 years). Based on clinical grading, patients were classified as moderate intermittent (*n* = 6) or moderate persistent VKC (*n* = 9). Disease duration ranged from acute onset (10 days) to over 10 years, with most persistent cases exhibiting symptoms for more than 3 years and frequent exacerbations. Seasonal triggers were reported in 67% of patients, while 33% showed a perennial course. The palpebral form was most common (47%), followed by mixed (33%) and limbal (13%) variants. Papillary hypertrophy was present in all patients; limbal papillae were observed in limbal and mixed subtypes. Horner–Trantas dots were noted in one patient, and superficial punctate keratitis in three. Ocular surface complications—including conjunctival scarring, corneal epithelial defects, and steroid-induced glaucoma—were more frequent in moderate persistent VKC (Table 1).

### 2.2. Differential Expression of Tear Cytokines in VKC

**Figure 2:**
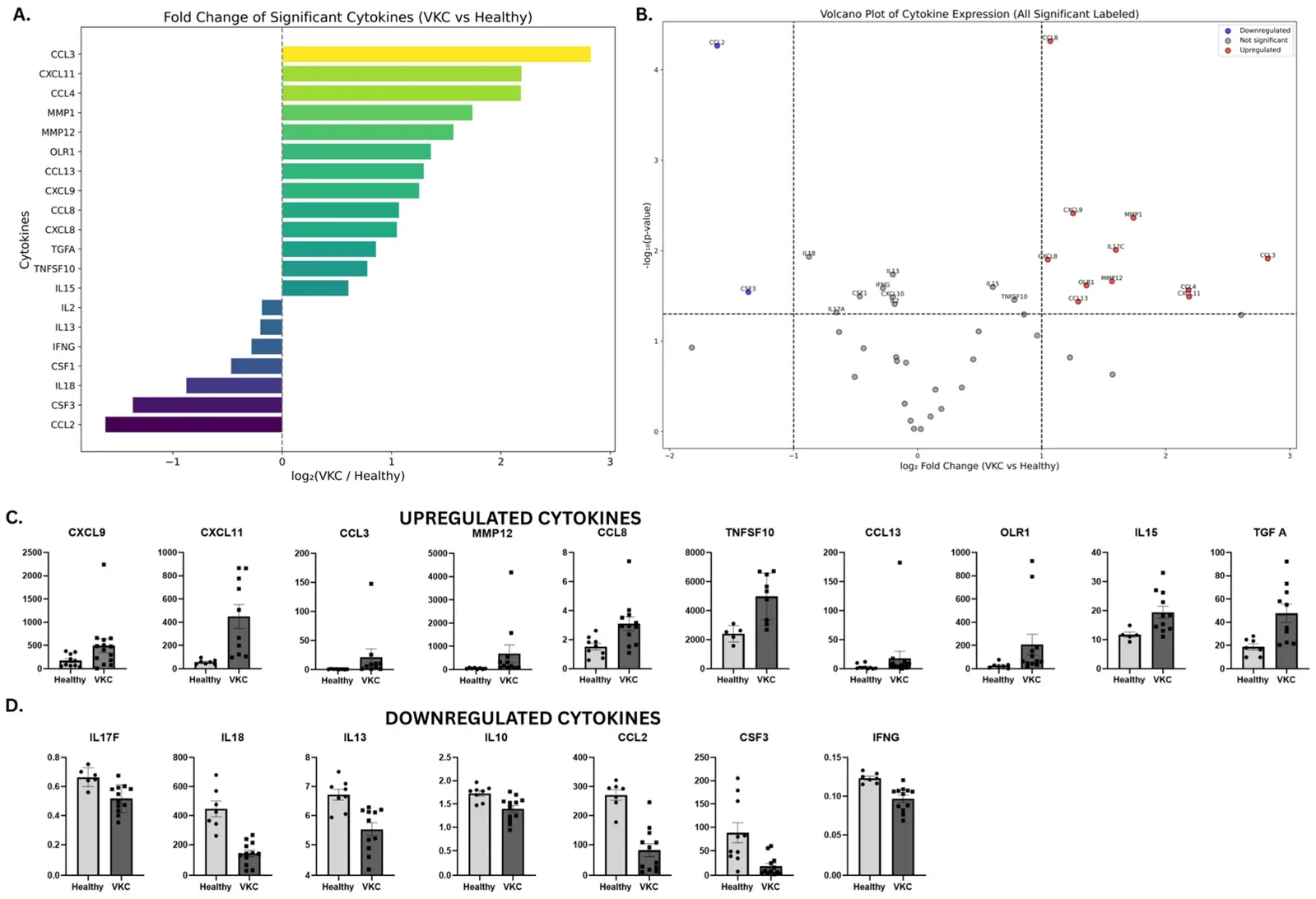
Tear cytokine profile in VKC compared to healthy controls. (**A**) Bargraph representing the fold change of significantly differentially expressed cytokines, showing marked upregulation of CCL3, CXCL11, MMP1, MMP12, OLR1, CCL13, IL15, CXCL8, TNFSF10, CXCL9, and others in VKC tears. (**B**) Volcano plot illustrating the distribution of cytokines based on fold change and statistical significance, highlighting both upregulated and downregulated targets. (**C**) Bar graphs depicting the expression levels of statistically significant upregulated cytokines, with CXCL9 (*p* = 0.0007), CCL13 (*p* = 0.0006), and CXCL8 (*p* = 0.0007) among the most significant. (**D**) Bar graph showing significantly downregulated cytokines, including CCL2 (*p* = 0.0003) and CSF3 (*p* = 0.0047), along with their expression range and significance.

**Figure A1:**
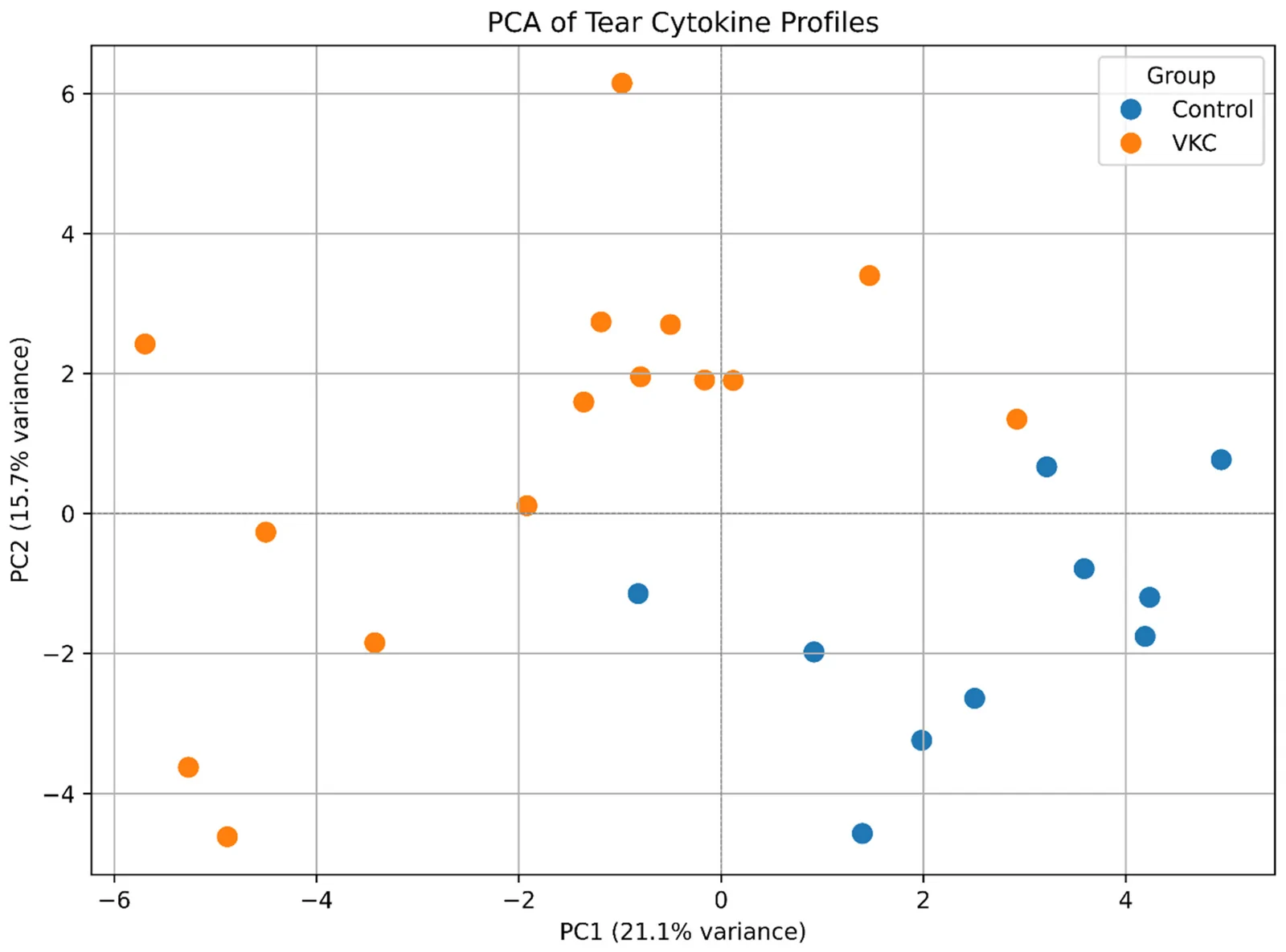
Principal Component Analysis (PCA) of tear cytokine profiles in VKC and healthy control subjects. Each point represents an individual sample, plotted by the first two principal components (PC1 and PC2), which together account for 36.8% of the total variance in the dataset. Orange points indicate VKC patients, while blue points represent healthy controls. The separation along PC1 and PC2 suggests distinct cytokine expression patterns between the two groups. Cytokine concentrations were normalized and scaled prior to PCA.

Tear cytokine profiling in VKC patients demonstrated a significant alteration in multiple inflammatory mediators compared to healthy controls (Figure A1). In this study, 45 cytokines were analyzed using the OLINK platform across 10 control and 15 VKC tear samples Of the 45 proteins included in the panel, 44 met the predefined detection and quality-control criteria. IL-4 was below the assay detection threshold and was therefore excluded from quantitative and comparative analyses. Among the 44 analyzable cytokines, 20 showed statistically significant differential expression (*p* < 0.05): 13 were upregulated and 7 were downregulated in VKC (Figure 2). Among the upregulated cytokines, Chemokine (C-C motif) ligand 3 (CCL3) showed the highest fold change (log_2_FC = 2.82, *p* = 0.0157), followed by CXCL11 (log_2_FC = 2.18, *p* = 0.0116), CCL4 (log_2_FC = 2.18, *p* = 0.0454), MMP1 (log_2_FC = 1.74, *p* = 0.0079), and MMP12 (log_2_FC = 1.57, *p* = 0.0239). Other significantly elevated cytokines included CXCL9, CCL13, CXCL8, TNFSF10, OLR1, TGF-α, and IL15. In contrast, CCL2 was the most significantly downregulated (log_2_FC = –1.62, *p* = 0.0001), along with CSF3 (log_2_FC = –1.37, *p* = 0.0456), IL18, CSF1, and IFNG. These findings highlight a distinct inflammatory signature in VKC tears involving immune cell recruitment and tissue remodelling.

### 2.3. Functional Enrichment and Network Analysis of VKC-Associated Cytokines

**Figure 3:**
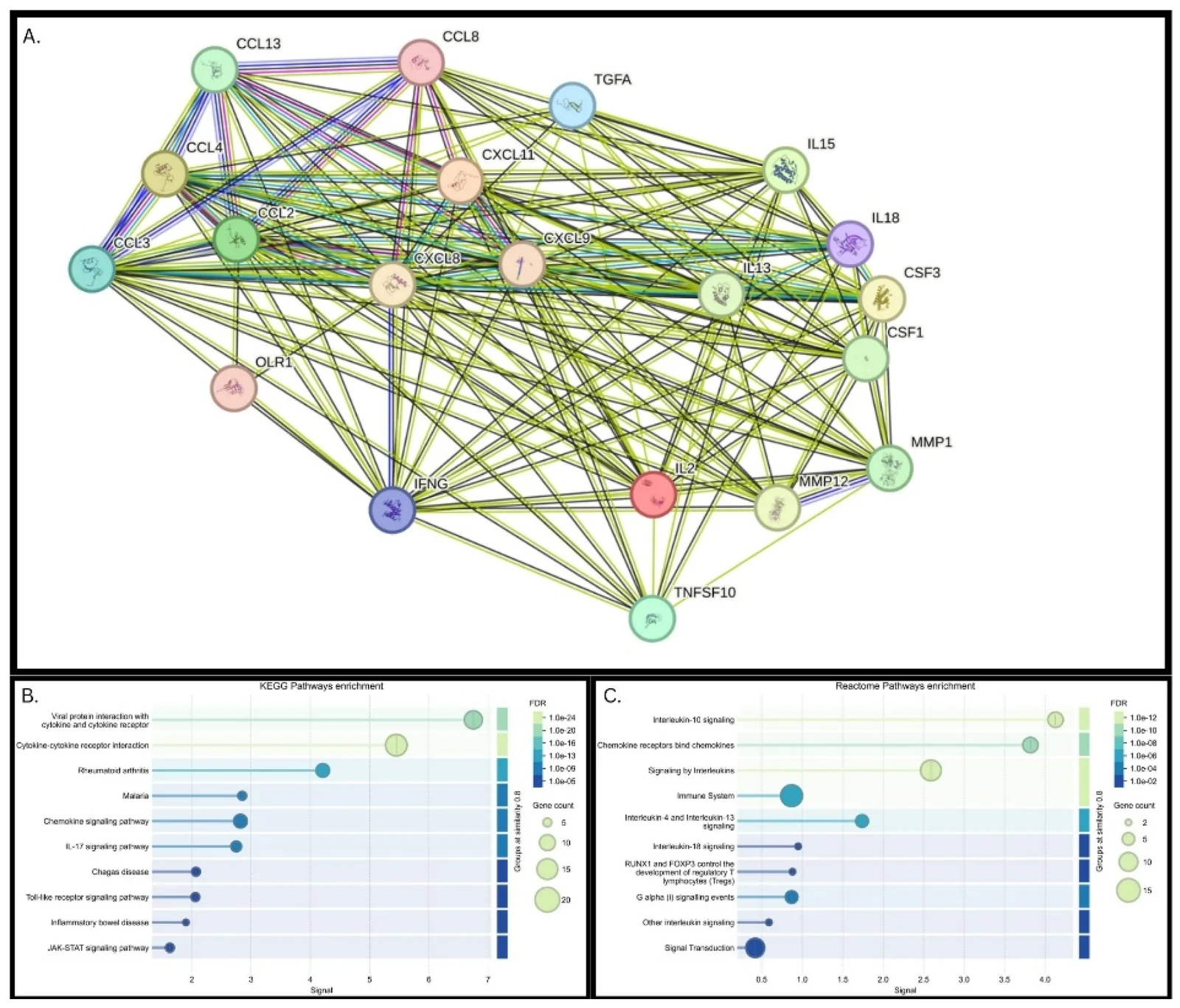
Network interaction and enrichment analysis of differentially expressed tear cytokines in VKC. (**A**) STRING network visualization of significantly dysregulated tear cytokines in VKC patients, showing protein–protein interactions with high confidence scores. Prominent nodes include CXCL8, TNFSF10, CCL3, CXCL11, CXCL9, IL15, MMP1, and MMP12. (**B**) KEGG pathway enrichment analysis highlighting cytokine–cytokine receptor interaction, chemokine signaling, IL-17 signaling, and JAK-STAT signaling pathways. (**C**) Reactome pathway enrichment indicating significant involvement in interleukin signaling, chemokine receptor activity, and immune system modulation.

**Figure A3:**
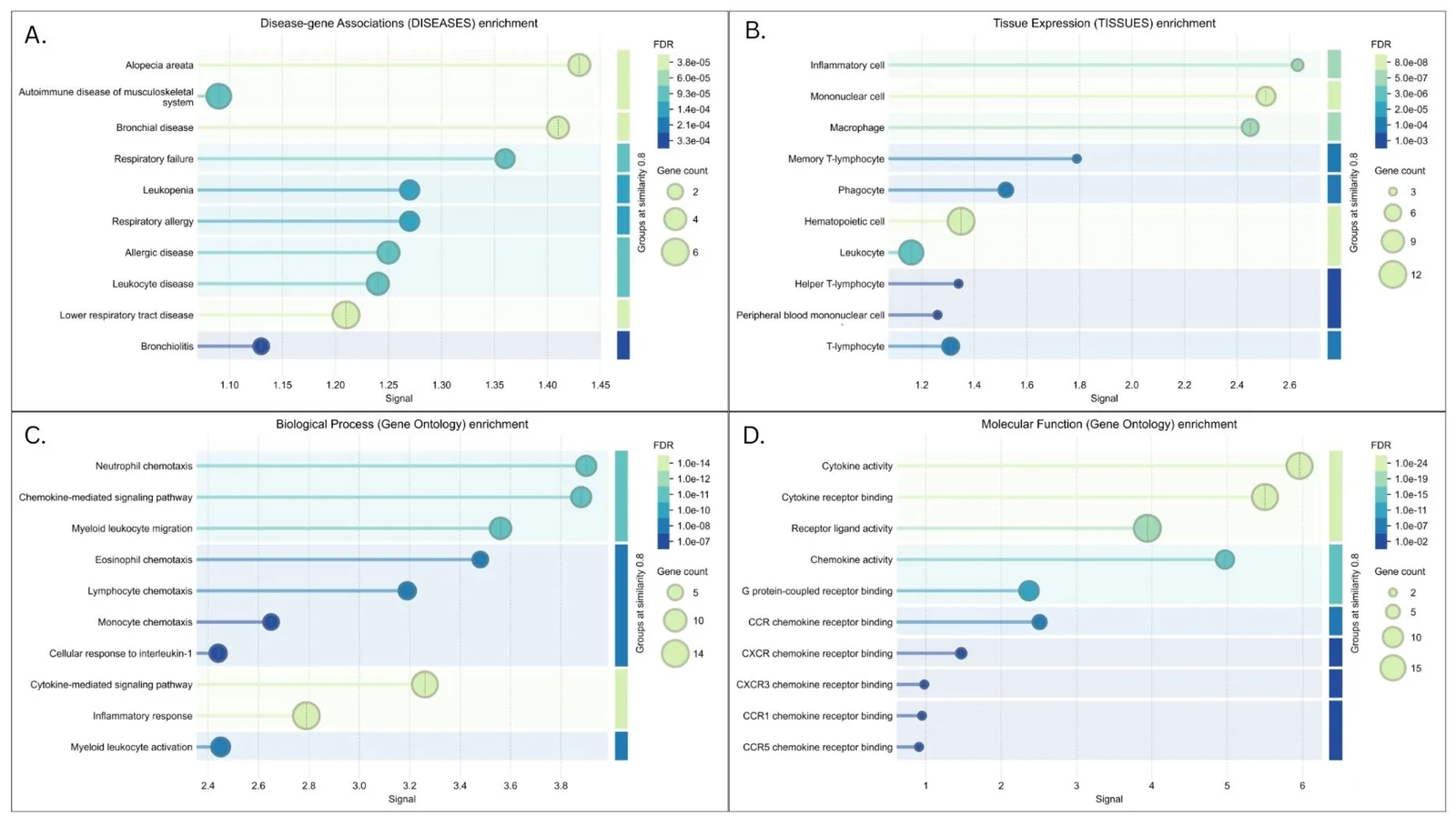
Functional enrichment analysis of differentially expressed tear cytokines in vernal keratoconjunctivitis. (**A**) Disease–gene association enrichment showing significant links with allergic, inflammatory, autoimmune, and respiratory disorders, including alopecia areata, autoimmune musculoskeletal disease, bronchial disease, respiratory failure, leukaemia, respiratory allergy, allergic disease, and bronchitis. (**B**) Tissue and cell-type expression enrichment demonstrating predominant association with inflammatory cells, mononuclear cells, macrophages, memory T lymphocytes, phagocytes, haematopoietic cells, leukocytes, natural killer cells, peripheral blood mononuclear cells, and T lymphocytes. (**C**) Gene Ontology biological process enrichment highlighting neutrophil chemotaxis, chemokine-mediated signalling, myeloid and lymphocyte migration, eosinophil chemotaxis, inflammatory responses, cytokine-mediated signalling, and myeloid leukocyte activation. (**D**) Gene Ontology molecular function enrichment showing significant cytokine activity, cytokine receptor binding, receptor–ligand activity, chemokine activity, G protein-coupled receptor binding, and binding to CCR and CXCR chemokine receptors. Bubble size represents the number of associated genes, bubble colour indicates the false discovery rate, and the horizontal axis represents the enrichment signal.

STRING network analysis (Figure 3A) revealed a dense protein–protein interaction network among the significantly dysregulated cytokines, highlighting key hubs such as CXCL8, CXCL9, IL15, TNFSF10, and MMP1. These genes are known to be involved in tightly connected inflammatory and chemokine signaling pathways. KEGG pathway enrichment (Figure 3B) demonstrated significant involvement of cytokine–cytokine receptor interaction, IL-17 signaling, JAK-STAT signaling, and Toll-like receptor pathways, all known contributors to allergic and chronic inflammatory responses. Reactome pathway enrichment (Figure 3C) supported these findings by indicating significant enrichment in interleukin signaling, chemokine receptor binding, and innate immune system activation. Disease-gene enrichment analysis (Figure A3A) further associated these cytokines with asthma, allergic diseases, bronchitis, and autoimmune conditions, reflecting the overlapping pathology of VKC. Tissue-specific enrichment (Figure A3B) revealed these cytokines to be predominantly expressed in inflammatory and myeloid-derived immune cells such as monocytes, macrophages, and T cells. GO Biological Process enrichment (Figure A3C) highlighted neutrophil chemotaxis, myeloid leukocyte activation, and inflammatory response as key biological events. Molecular Function analysis (Figure A3D) emphasized cytokine activity and chemokine receptor binding, specifically involving CCR1, CCR5, and CXCR3, known mediators of eosinophil and lymphocyte recruitment in allergic conjunctivitis.

### 2.4. Cytokine Shifts and Clinical Associations in VKC Subtypes

**Figure 4:**
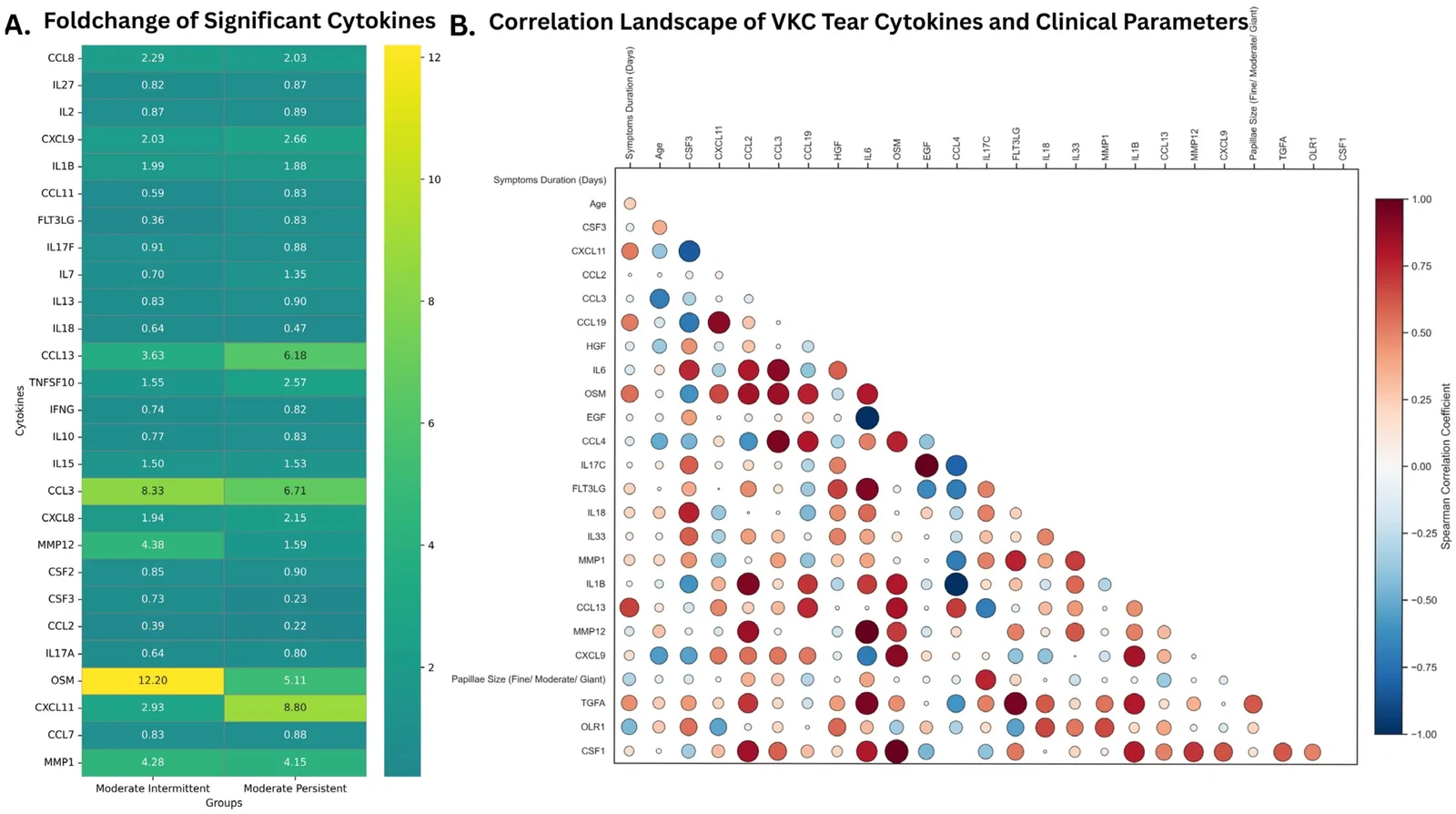
Comparative analysis of significant tear cytokines in VKC subgroups and their correlation with clinical features. (**A**) Heatmap displaying fold change values of the same cytokines in moderate intermittent and moderate persistent VKC groups relative to healthy controls, highlighting elevated fold changes particularly for OSM, CCL3, and CXCL11 in moderate persistent cases. (**B**) Correlogram illustrating Spearman’s correlation between cytokine levels and clinical parameters including symptom duration, limbal reaction, SPK score, and papillae size. Circle size and color intensity indicate the strength and direction of correlation.

Fold change analysis comparing VKC subtypes to healthy controls revealed that several cytokines were significantly upregulated in the persistent VKC group. Notably, CXCL11 exhibited the most prominent increase with a log_2_ fold change of 2.72 (*p* = 0.0186), followed by Oncostatin M (OSM) (log_2_FC = 2.21, *p* = 0.0295), MMP1 (log_2_FC = 1.72, *p* = 0.0320), CCL13 (log_2_FC = 1.63, *p* = 0.0211), and CXCL9 (log_2_FC = 1.42, *p* = 0.0006). These changes suggest enhanced chemotactic signaling and extracellular matrix remodeling in the persistent VKC subtype. While some of these cytokines also showed elevated expression in the intermittent group (e.g., OSM log_2_FC = 3.09, MMP1 log_2_FC = 1.77), their upregulation was more pronounced in the persistent form. The data highlight a distinct pattern of progressive immune activation and matrix degradation in VKC, with persistent cases exhibiting stronger cytokine shifts from baseline tear profiles observed in healthy individuals (Figure 4). Spearman correlation analysis of the top 25 variables revealed that CXCL9, MMP12, CCL13, IL1B, and MMP1 showed strong positive correlations with papillae size. CXCL11, OSM, and IL6 were positively correlated with both symptom duration and papillae size, while CSF3, IL18, and EGF showed variable or negative correlations with these clinical parameters. Notably, CCL19, CCL4, and IL6 formed a cluster of inter-correlated cytokines. The pattern of correlations indicates distinct cytokine–clinical associations, particularly involving chemokines, matrix-modulating enzymes, and interleukins in VKC.

## 3. Discussion

Vernal keratoconjunctivitis (VKC) is a chronic allergic disorder of the ocular surface that predominantly affects children and adolescents and poses a substantial clinical burden in tropical regions such as India. While many patients initially present with seasonal, self-limiting disease, a clinically important subset progresses to a moderate persistent phenotype characterized by chronic inflammation, reduced therapeutic responsiveness, and a heightened risk of conjunctival fibrosis and corneal complications. Understanding the immunological changes that accompany this transition is critical for improving disease stratification and guiding targeted interventions. In this study, we provide a comprehensive tear-based immune profile of VKC in an Indian cohort and demonstrate that disease persistence is associated with a cytokine landscape that extends beyond classical Th2-driven allergy [1,2,5,31].

**Figure 5:**
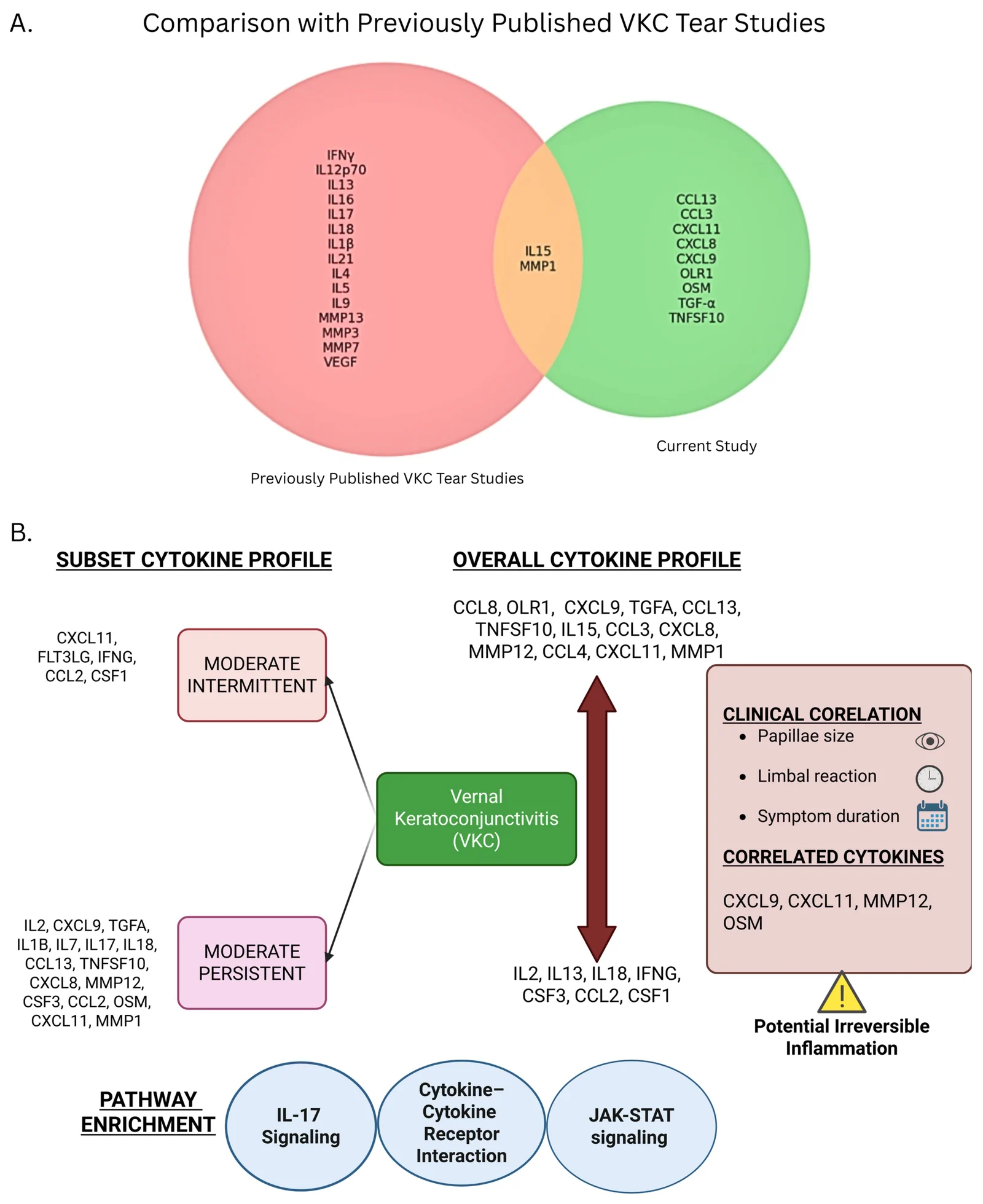
Literature-based contextualization and summary of tear cytokine changes in VKC. (**A**) Descriptive comparison of mediators identified in the present VKC cohort with cytokines and matrix-associated proteins previously reported in independent VKC tear studies. This comparison is literature-based and does not represent a direct ethnic, geographical, or matched-cohort analysis. Differences should be interpreted in view of variations in clinical characteristics, sample collection, treatment status, assay platforms, and analyte coverage. (**B**) Schematic summary of cytokine profiles observed in moderate intermittent and moderate persistent VKC and their associations with papillary size, limbal involvement, and symptom duration.

Tear cytokine profiling revealed a marked increase in inflammatory mediators in VKC patients compared to healthy controls, with significant upregulation of CCL3, CXCL9, CXCL11, MMP1, and MMP12. These molecules are centrally involved in leukocyte recruitment, chemotaxis, and extracellular matrix remodelling—processes that closely mirror the clinical features observed in VKC, including papillary hypertrophy, limbal thickening, and chronic conjunctival inflammation. Importantly, stratification of patients into moderate intermittent and moderate persistent subtypes uncovered distinct immunological differences associated with disease severity. Patients with moderate persistent VKC exhibited substantially higher levels of CXCL11, MMP1, and OSM, indicating a sustained, tissue-damaging inflammatory response rather than episodic allergic flares. An important observation was that IL-4 was below the detection threshold of the OLINK assay in the tear samples analysed in this cohort. This finding should be interpreted cautiously and specifically within the tear-film compartment and analytical conditions of the present study. It does not establish the absence of IL-4 activity in conjunctival tissue and does not exclude local IgE-dependent or Th2-associated mechanisms [1,2,5,20,29]. Differences from earlier studies may reflect variation in disease stage, clinical phenotype, treatment history, biological compartment, sample-processing procedures, assay platform, or detection threshold [3,32,33]. Nevertheless, the prominent elevation of CXCL9, CXCL11, CCL3, MMP1, MMP12, and OSM indicates that established VKC involves additional chemokine-mediated and tissue-remodelling pathways beyond the classical allergic response [34,35]. Among these mediators, OSM emerged as a particularly strong marker of disease persistence. Notably, the magnitude and consistency of OSM elevation across persistent cases distinguished it from other inflammatory mediators, suggesting a central role in sustaining chronic ocular surface pathology. OSM is well recognized for its role in chronic inflammation, fibrosis, epithelial barrier dysfunction, and stromal activation, and its elevation in persistent VKC aligns closely with the clinical phenotype commonly observed in Indian patients—namely, perennial symptoms, corneal involvement, and conjunctival scarring [36]. These findings suggest that OSM may serve both as a biomarker of disease chronicity and a potential therapeutic target [34,35,36,37,38,39]. Comparison with previously published VKC tear studies demonstrated both overlapping and distinct inflammatory mediators. The cytokine profiles attributed to Italian VKC cohorts were derived from independent published studies and were not generated or reanalysed as part of the present investigation. The comparison is therefore descriptive and literature-based rather than a direct population or ethnic comparison (Figure 5A). Differences between the reported profiles may reflect variation in clinical phenotype, disease severity, treatment history, environmental exposure, tear-collection procedures, analytical platforms, analyte coverage, and detection thresholds. Consequently, the current findings should not be interpreted as demonstrating an ethnicity-dependent immune profile. Instead, they provide a tear-based cytokine profile from the present Indian cohort that can be compared with and validated across future independent populations [38,39,40]. A key strength of this study is the use of OLINK^®^ proximity extension assay–based tear proteomics, which enabled highly sensitive, multiplex detection of low-abundance cytokines from minimal tear volumes. This platform overcomes many limitations of earlier VKC studies, including restricted analyte coverage and insufficient sensitivity, and allows unbiased pathway-level analysis of the ocular surface immune environment. The ability to capture complex immune signatures from non-invasive tear samples is particularly advantageous in pediatric populations and supports the translational potential of this approach (Appendix A—Table A1). Although the present study employed a targeted tear-proteomic approach, integration with complementary omics platforms could provide a more comprehensive understanding of VKC pathogenesis. Combining tear proteomics with transcriptomics may help identify the cellular sources and transcriptional regulation of dysregulated inflammatory mediators, while metabolomics and lipidomics could reveal alterations in oxidative stress, energy metabolism, and lipid-derived inflammatory signalling. Integration with ocular-surface microbiome profiling and detailed clinical phenotyping may further identify molecular subgroups associated with disease persistence, severity, and treatment response. Multi-omics approaches, supported by computational modelling and artificial-intelligence-assisted data integration, have increasing potential for biomarker discovery, patient stratification, prediction of therapeutic response, and personalized treatment development. Therefore, the cytokine profile identified in the present study may serve as an initial molecular framework for future multi-omics investigations in larger, longitudinal VKC cohort [40].

Several limitations should be acknowledged. First, the modest sample size, particularly after stratification into moderate intermittent and moderate persistent VKC, limits statistical power and the generalizability of the subgroup findings. The results should therefore be considered exploratory and require validation in larger independent cohorts. Second, the cross-sectional design does not establish whether the identified cytokine changes precede disease persistence or reflect established inflammation. Third, total and allergen-specific tear IgE were not measured because immunoglobulins are not included in the predefined OLINK Target 48 Cytokine Panel and IgE assessment was outside the original objective and analytical workflow of the study. Therefore, direct relationships between local IgE-mediated sensitization and the observed cytokine profile could not be evaluated. Fourth, the comparison with previously published VKC cohorts was literature-based and was limited by differences in patient characteristics, sample collection, treatment status, analytical platforms, and analyte coverage. Finally, functional experiments were not performed, and the biological roles of OSM, CXCL11, and matrix metalloproteinases in VKC require investigation in future mechanistic studies (Table A2).

Overall, this study provides a tear-based cytokine profile of VKC in a clinically characterized Indian cohort (Figure 5A). The results demonstrate prominent dysregulation of chemokines and tissue-remodelling mediators, particularly in moderate persistent disease. The finding that IL-4 was below the assay detection threshold does not exclude IgE-dependent or Th2-associated mechanisms but indicates that additional inflammatory pathways are detectable in established VKC. OSM, CXCL9, CXCL11, MMP1, and MMP12 warrant further investigation as candidate markers of disease chronicity and severity. These exploratory findings require validation in larger, longitudinal, and independent cohorts before clinical or therapeutic application (Figure 5B).

## 4. Materials and Methods

### 4.1. Study Design and Participants

This was a cross-sectional observational study conducted at Dr. Shroff’s Charity Eye Hospital (SCEH), New Delhi. The study was approved by the Institutional review Board (IRB) of SCEH and a total of 15 patients were enrolled. VKC diagnosis was based on characteristic clinical features including giant papillae, limbal involvement, and corneal changes, assessed by slit-lamp microscopy. Classification into moderate or intermittent VKC was done according to symptom severity and frequency of exacerbations. Healthy controls were age- and sex-matched individuals without any history of ocular surface disease, allergy, or systemic inflammatory conditions. All participants provided written informed consent.

### 4.2. Clinical Data Collection

Clinical parameters such as symptom severity, disease duration, presence and extent of papillary hypertrophy, limbal changes, and corneal manifestations (including shield ulcers and superficial punctate keratitis) were documented during ophthalmic evaluations. Patients were categorized based on symptoms into intermittent and persistent forms, aligning with standard classifications typically describing seasonal (intermittent) and perennial (persistent) clinical presentations. Disease severity grading followed the Bonini classification, a recognized system incorporating clinical parameters like conjunctival inflammation, papillary size, and corneal involvement to classify VKC severity into mild, moderate, and severe stages. To minimize the potential influence of ongoing therapies, all subjects had discontinued any systemic and topical treatments, including corticosteroids, cyclosporine, tacrolimus, and antihistamines, at least two weeks before tear sample collection.

### 4.3. Tear Sample Collection and Storage

Tear samples were collected from 15 patients, including 6 with moderate intermittent and 9 with moderate persistent VKC. Samples were collected from the singular, non-medicated eye of each patient using Schirmer’s strips placed in the lower conjunctival fornix for 5 min. Immediately after collection, the strips were transferred into sterile RNase/DNase-free microcentrifuge tubes and stored at −80 °C until further processing.

### 4.4. Tear Protein Isolation and Quantification

Stored Schirmer’s strips were thawed on ice and eluted in 60 μL of Phosphate-Buffered Saline (PBS), pH 7.4 (Himedia laboratories Pvt Ltd., Maharashtra, India Cat No. M1866) supplemented with cOmplete^™^ Protease Inhibitor Cocktail (Hoffmann-La Roche AG, Basel, Switzerland Catalogue No. 11697498001). The strips were vortexed and mechanically crushed on ice multiple times to enhance protein recovery. Eluates were then centrifuged, and total protein concentration was quantified using the Pierce™ BCA Protein Assay Kits (Thermo Scientific, Rockford, IL, USA, Catalogue no. 23227) following standard protocols.

### 4.5. Cytokine Profiling Using OLINK Proteomics

Eluted tear proteins were analyzed using the OLINK^®^ Target 48 Cytokine Panel [30] (OLINK Proteomics, Uppsala, Sweden), which quantifies 45 inflammation-related proteins using Proximity Extension Assay (PEA) technology. Protein detection was based on proximity extension assay technology. In this assay, each target protein is recognized by a pair of target-specific antibodies conjugated to complementary oligonucleotide sequences. Binding of both antibodies to the same target brings the oligonucleotides into proximity, allowing them to hybridize and undergo enzymatic extension. This generates an analyte-specific DNA reporter sequence that is subsequently amplified and quantified. Protein abundance was reported as Normalized Protein Expression values on a log2 scale. Internal controls were included to monitor incubation, extension, amplification, detection, background signal, and technical variation. Data normalization and quality-control assessment were performed according to the manufacturer’s standard analytical workflow. NPX values were used for statistical comparisons, clustering, principal-component analysis, heatmap generation, and correlation analyses. Absolute concentration values, where available, were used for descriptive fold-change calculations and selected graphical representations. Proteins that did not meet the predefined assay detection or quality-control criteria were excluded from downstream analysis. IL-4 was below the assay detection threshold and was therefore excluded from quantitative comparisons [29,31].

### 4.6. Bioinformatic and Statistical Analysis

All statistical and bioinformatic analyses were conducted using Jupyter Notebook (version 7.6.0; Project Jupyter) with standard Python packages 3.0.4 (pandas 3.0.5, NumPy 2.5.1, SciPy 1.18.0) for data manipulation, statistical computation, and visualization. Outliers were identified and removed using the interquartile range (IQR) method. After normalization, fold changes were calculated for all cytokines by comparing VKC samples to healthy controls. Unpaired *t*-tests were used to assess statistical significance, with *p* < 0.05 considered significant. While statistical analyses were conducted in Python, GraphPad Prism (GraphPad Prism 10.5.0) [32] was used to generate bar graphs of absolute cytokine values for clearer visual presentation.

To represent differential expression, volcano plots were generated based on calculated log2 fold changes and corresponding *p*-values. Heatmaps were created to compare cytokine expression profiles between VKC and healthy individuals, and further stratified to compare moderate intermittent and moderate persistent VKC subtypes, highlighting subgroup-specific differences. A Spearman correlation-based correlogram was constructed to explore associations between cytokine levels and clinical parameters such as symptom duration, papillae size, and clinical severity score. To assess biological relevance, significantly dysregulated cytokines were analyzed using STRING for pathway enrichment [33], identifying associated KEGG pathways, Reactome pathways, and disease linkages. All visualization outputs—including volcano plots, heatmaps, PCA, and correlation matrices—were used to support interpretation of the cytokine profiling results.

## 5. Conclusions

This study provides a comprehensive overview of tear cytokine dysregulation in VKC, identifying distinct molecular signatures that differentiate disease subtypes and correlate with clinical severity. In this Indian cohort, persistent VKC is characterized by a tear cytokine profile dominated by chemokines and tissue-remodeling mediators, including CXCL11, MMP1, and OSM, reflecting a shift toward chronic, self-sustaining inflammation and increased risk of irreversible tissue damage. Notably, the absence of detectable IL-4 challenges the classical Th2-centric paradigm of VKC pathogenesis and suggests that alternative immune pathways drive established disease. These findings underscore important population-specific differences from Western cohorts and highlight the need to tailor therapeutic strategies to the underlying immunological landscape of Indian patients. By redefining disease mechanisms and identifying biomarkers of chronicity, OLINK-based tear proteomics emerges as a powerful, non-invasive approach to advance precision diagnosis, guide targeted therapy, and inform future translational research in ocular allergy.

## Figures and Tables

**Table 1:** Clinical details of VKC patients including grade, age, symptom duration, allergy type, ocular findings. Patient demographic and clinical details for the VKC study cohort. VKC, Vernal Keratoconjunctivitis; M, Male; F, Female; BE, Both Eyes; RE, Right Eye; LE, Left Eye; C, Congestion; P, Papillae; S, Scarring; Mod., Moderate; N, Normal (no significant findings); Mixed, both limbal and palpebral involvement.

Patient	Grade	Age	Symptoms Duration	Ocular Allergy (Perennial/Seasonal)	Lids (Congestion/Papillae/Scarring/Blepharitis)	Papillae Size (Fine/Moderate/Giant)	VKC Type (Limbal/Palpebral/Mixed)
Patient 1	Moderate intermittent	9/M	1 week	Seasonal	BE: C, P	BE: Fine	Mixed
Patient 2	Moderate Intermittent	27/F	1 year	Seasonal	RE: P, LE: P, S	BE: Fine	Mixed
Patient 3	Moderate Intermittent	13/F	3 months	Perennial	BE: C, P	BE: Giant	Palpebral
Patient 4	Moderate intermittent	11/F	3 years	Seasonal	P	Moderate	Palpebral
Patient 5	Moderate Intermittent	9/M	10 days	Seasonal	BE: C, P	RE: Mod. LE: Fine	Palpebral
Patient 6	Moderate Intermittent	9/M	10 days	Seasonal	BE: C, P	RE: Mod. LE: Fine	Palpebral
Patient 7	Moderate Persistent	13/M	2 years	Perennial	N	N	Limbal
Patient 8	Moderate Persistent	8/M	3 years	Seasonal	P	Fine	Mixed
Patient 9	Moderate Persistent	25/M	15 years	Seasonal	BE: C, P	BE: Fine	Palpebral
Patient 10	Moderate Persistent	25/M	15 years	Seasonal	BE: C, P	BE: Fine	Palpebral
Patient 11	Moderate Persistent	8/M	3 years	Seasonal	P	Fine	Mixed
Patient 12	Moderate Persistent	8/M	3 years	Seasonal	P	Fine	Mixed
Patient 13	Moderate Persistent	13/M	2 years	Perennial	N	N	Limbal
Patient 14	Moderate Persistent	13/M	3 years	Perennial	C, P	Giant	Palpebral
Patient 15	Moderate Persistent	18/M	10+ years	Perennial	BE: C, P	BE: Fine	Palpebral

**Figure A2:**
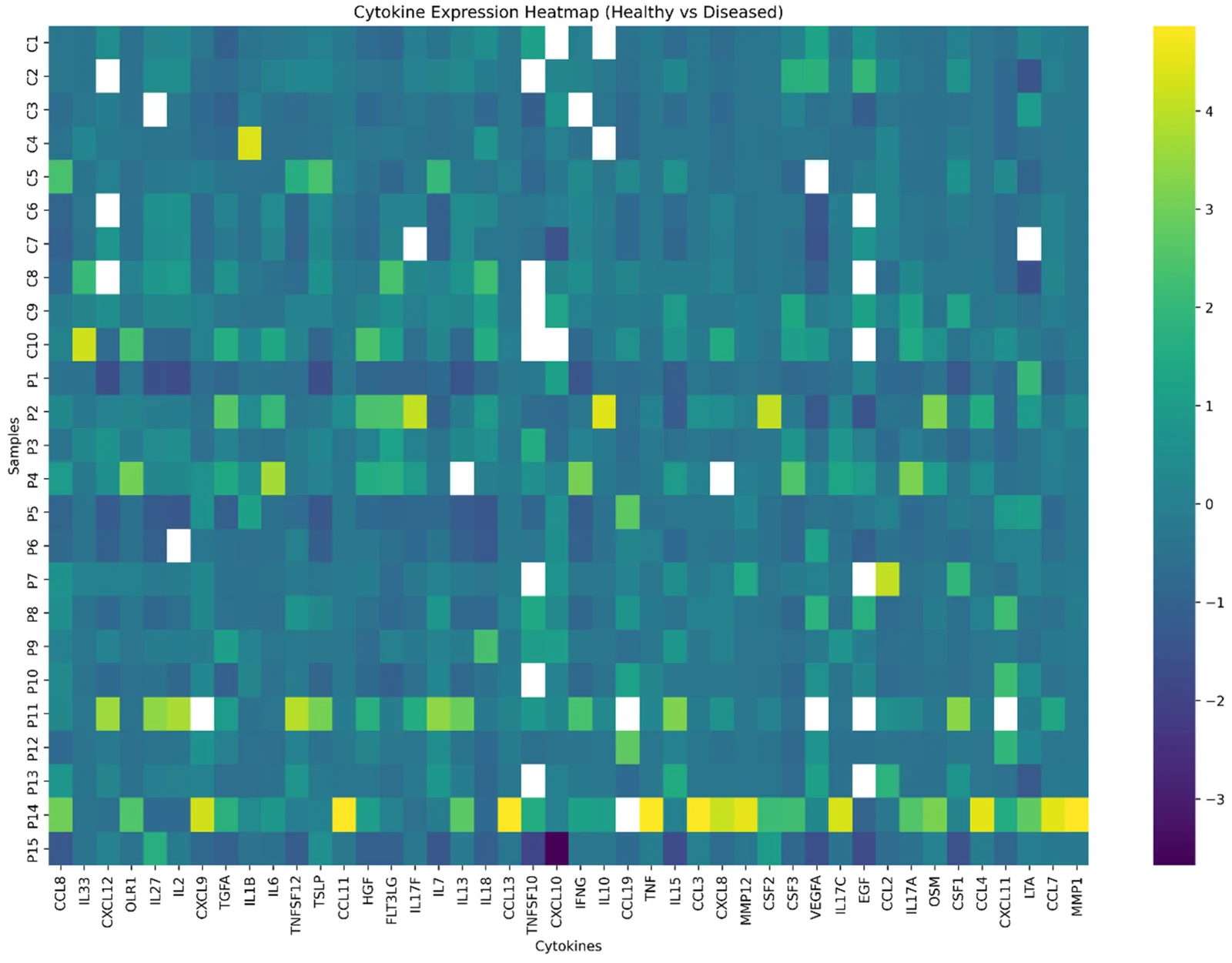
Heatmap of normalized tear cytokine expression across healthy controls (C1–C10) and VKC patients (P1–P15). Each row represents a sample, and each column represents an individual cytokine. Color intensity reflects the standardized expression level (z-score) of each cytokine, with yellow indicating higher and purple indicating lower expression. White blocks indicate missing or undetectable values. This visualization highlights inter-patient variability and differential expression of multiple cytokines between healthy and VKC groups.

## Data Availability

The data presented in this study are available within the article. Additional data may be available from the corresponding author upon reasonable request, subject to institutional and ethical restrictions.

## Abbreviations

The following abbreviations are used in this manuscript:

VKC	Vernal Keratoconjunctivitis
OSM	Oncostatin M
MMP	Matrix Metalloproteinase
CCL	C-C Motif Chemokine Ligand
CXCL	C-X-C Motif Chemokine Ligand
IL	Interleukin
TNF	Tumor Necrosis Factor
EGF	Epidermal Growth Factor
VEGFA	Vascular Endothelial Growth Factor A
OLINK	Olink Proteomics Platform
PEA	Proximity Extension Assay
NPX	Normalized Protein Expression
PCA	Principal Component Analysis
IQR	Interquartile Range
IRB	Institutional Review Board
SPK	Superficial Punctate Keratitis

## Appendix A

**Table A1:** Comparative summary of tear cytokine profiles and clinical characteristics of VKC reported in Italian/Middle Eastern cohorts versus the present Indian cohort.

Feature	Italian/Middle Eastern VKC	Indian VKC (Present Study)
Dominant cytokines	IL-4, IL-5, IL-13 (Th2)	CCL3, CXCL9, CXCL11, MMP1, OSM
IL-4 detection	Consistently present	Not detected
OSM	Rarely reported	Significantly elevated
Clinical pattern	Seasonal, Th2-driven	Perennial, chronic, fibrosing

**Table A2:** Principal Immune Cell Sources and Functional Roles of Selected Cytokines, Chemokines, and Related Molecules.

Cytokine/Marker	Principal Immune Cell Sources	
CXCL9	Monocytes, macrophages, endothelial cells, fibroblasts; Th1 cells, NK cells, NKT cells, CD8+ T cells	Induced by IFN-γ; attracts cytotoxic lymphocytes and NK cells
CXCL11	Monocytes, endothelial cells, fibroblasts, dendritic cells (DCs)	Attracts T cells (CD4+, CD8+), NK cells, and dendritic cells; involved in antitumor immunity
CCL3	Monocytes, macrophages, dendritic cells, activated T cells (CD4+, CD8+)	Key in recruiting and activating T cells, B cells, dendritic cells, and NK cells; prominent in inflammatory sites
MMP12	Macrophages, monocytes, dendritic cells	Functions as an antigen-presenting cell–associated molecule, also involved in matrix remodeling and immune activation
CCL8	Macrophages, monocytes, intestinal epithelial cells, podocytes	Stimulates recruitment of T cells, B cells, NK cells, other leukocytes to inflammatory tissues
CCL13	Monocytes, eosinophils, T cells, immature dendritic cells	Promotes migration of eosinophils, monocytes, T cells, and DCs; upregulated in allergic and inflammatory responses
OLR1 (LOX-1)	Endothelial cells, macrophages, smooth muscle cells	Not a classic cytokine, but a receptor: mainly present on myeloid and vascular cells, upregulated in inflammation
IL-15	Dendritic cells, macrophages, monocytes, endothelial cells, stromal cells	Critical for growth and activation of NK cells, CD8+ T cells, γδ T cells through trans-presentation; not produced by T or NK cells
TGFα	Monocytes, macrophages, platelets, epithelial cells	Involved in wound healing and cellular proliferation; secreted at inflammatory sites
